# Higher criticism approach to detect rare variants using whole genome sequencing data

**DOI:** 10.1186/1753-6561-8-S1-S14

**Published:** 2014-06-17

**Authors:** Jing Xuan, Li Yang, Zheyang Wu

**Affiliations:** 1 Department of Mathematical Science, Worcester Polytechnic Institute, 100 Institute Road, Worcester, MA 01609-2280, USA

## Abstract

Because of low statistical power of single-variant tests for whole genome sequencing (WGS) data, the association test for variant groups is a key approach for genetic mapping. To address the features of sparse and weak genetic effects to be detected, the higher criticism (HC) approach has been proposed and theoretically has proven optimal for detecting sparse and weak genetic effects. Here we develop a strategy to apply the HC approach to WGS data that contains rare variants as the majority. By using Genetic Analysis Workshop 18 "dose" genetic data with simulated phenotypes, we assess the performance of HC under a variety of strategies for grouping variants and collapsing rare variants. The HC approach is compared with the minimal *p*-value method and the sequence kernel association test. The results show that the HC approach is preferred for detecting weak genetic effects.

## Background

Whole genome sequencing (WGS) is able to reveal complete genetic variations across the entire genome. It is deemed as the hope of decoding the mystery of genetic pathology to complex traits [1]. Comparing with single-variant tests in WGS, association tests for grouped variants potentially provide higher power for detecting genetic factors associated with diseases. First, because of small minor allele frequency (MAF), the association of a single rare variant is likely weak and unreliable [2]. One solution is to group and collapse the genotype data of rare variants [3]. Second, simultaneous analysis of multiple variants could better reveal genetic factors that are jointly functional for the biological mechanism of complex traits. Third, group-based tests are statistically attractive. In fact, from the multiple hypotheses testing perspective, finding the existence of a signal somewhere in a group is much easier than finding its exact location [4]. Therefore, it is more promising to target the discovery of sets of variants rather than individual variants.

To design appropriate association tests, it is critical to understand the properties of the data and the putative genetic variants. After the "lower fruits" have been picked up, the remaining variants to be identified usually have weak statistical associations with the response, because of either low allelic effects or rare variations [5]. At the same time, only a small proportion of candidates in WGS are disease variants. For addressing such weak and sparse genetic effects, the higher criticism (HC) procedure [4] has been shown optimal in genome-wide association studies (GWAS) [6]. However, whereas GWAS data mostly contain common variants, WGS data mostly contain rare variants. To apply HC to WGS data, proper grouping and collapsing strategies must be applied before the associations are tested. In the meantime, the grouping and collapsing strategies could critically influence the performance of association tests, and different tests may prefer different strategies. By using WGS data, we systematically explored a variety of common grouping and collapsing strategies and found the recommended setups for HC. The power comparisons of HC with other methods indicate that HC is preferred for detecting weak genetic effects.

## Methods

### Association tests

The HC procedure was designed for multiple hypotheses testing [7] and can be applied to test whether genetic associations exist in a set of variants. Under the null hypothesis that there is no genetic association, the *p*-value from the association test of each genetic variant follows a uniform distribution. Let $p_{\left(1\right)}\leq \ldots \leq p_{\left(n\right)}$ be the ordered *p*-values from *n *individual variants. Under the null, $p_{\left(i\right)}$ has mean *i/n*. As a maximum of the normalized empirical process, the HC statistic measures how much the *p*-values depart from the uniform distribution:

$$HC_{n}=max_{1\leq i\leq \frac{n}{2},\;p_{\left(i\right)}\geq 1/n}\frac{\sqrt{n}\left(i/n-p_{\left(i\right)}\right)}{\sqrt{p_{\left(i\right)}\left(1-p_{\left(i\right)}\right)}}.$$

Because the HC statistic uses a set of *p*-values as input, it is readily applicable for detecting genetic associations for either quantitative or binary traits in either population-based or family studies as long as the corresponding *p*-values are appropriately obtained. Here we used the two-tailed *p*-values to accommodate two directions of genetic effects. In analyzing the GAW18 data, the original genotype of common variants and the collapsed genotypes of rare variants (see later discussion) were applied to get *p*-values and thus the HC statistic.

The sequence kernel association test (SKAT) is a supervised test for the association between genetic variant groups and a continuous or dichotomous trait while accounting for covariates [8]. SKAT provides different weighting strategies for variants. However, here we chose the flat weight to fairly compare it with HC, for which genotypes are collapsed by the equal-weight sum. This "plain" setup helps us understand the statistical foundation of these two tests. In analyzing the GAW18 data, we applied the R package SKAT [9], and the original genotype data for both rare and common variants were used as the input of this package.

In the minimum *p*-value method (minP), each individual variant within a group is tested, and the smallest *p*-value is used to measure the significance of the group.

### Grouping and collapsing strategies

We considered four strategies to group variants according to fixed genome windows with lengths 10, 100, or 500 kbps and functional genes from the UCSC Known Gene [10]. The advantage of the fixed window grouping is that there is no overlap among the groups, the windows fully cover the whole genome, and the number of variants in the groups is more evenly distributed than those groups by genes. The advantage of gene grouping is that functional variants are likely concentrated in coding regions of the genome, which actually motivated exome-sequencing studies. However, there are difficulties caused by inconsistent definitions of genes, varieties of transcript segments and splicing sites, significantly different sizes of genes, and overlaps among genes. The largest drawback of gene-based grouping is that it ignores the majority of the genome that does not contain any genes. It is quite possible that disease variants or mutations could be located outside of genes.

A variant is defined as rare if its MAF is less than a threshold. Here the MAFs were estimated by the proportion of the minor alleles among the total observed alleles. We considered either 0.01 or 0.05 as the threshold. Within each window group, the rare variants allocated between any adjacent common variants were collapsed by two strategies: equal-weight sum or Madsen-Browning (MB) weight sum [3] of the original genotypes. The MB weight emphasizes the rare variants based on the rare variant-common disease model [11].

### Permutation test

For HC and minP, a permutation test is needed to accommodate the different variant numbers and correlation structures within different genome segment windows. Specifically, for each genome segment window, 1000 permutations of the genotype data were made, and test statistics were calculated. The empirical *p*-value for a group window is the proportion of the permutation statistics that are equal or more extreme than the corresponding statistic calculated from data.

## Results

Considering population-based studies, we included 142 independent individuals who have no missing genotype to the data analysis. We used the WGS dose files of chromosomes 1, 3, and 5 as the genotype data and the systolic blood pressure (SBP) as the phenotype. We considered two concepts of statistical power. First, the power indicates the ability of detecting overall true associations. It was estimated by the true positive rate of true association windows. Second, the power measures the ability of detecting a specific variant group with a specific genetic effect pattern. It was estimated by the true positive rate of the 200 simulation replicates. The knowledge of the simulated true variants was only used for evaluating the power of the association test, not for the design of the tests and data analysis.

**Figure 1:**
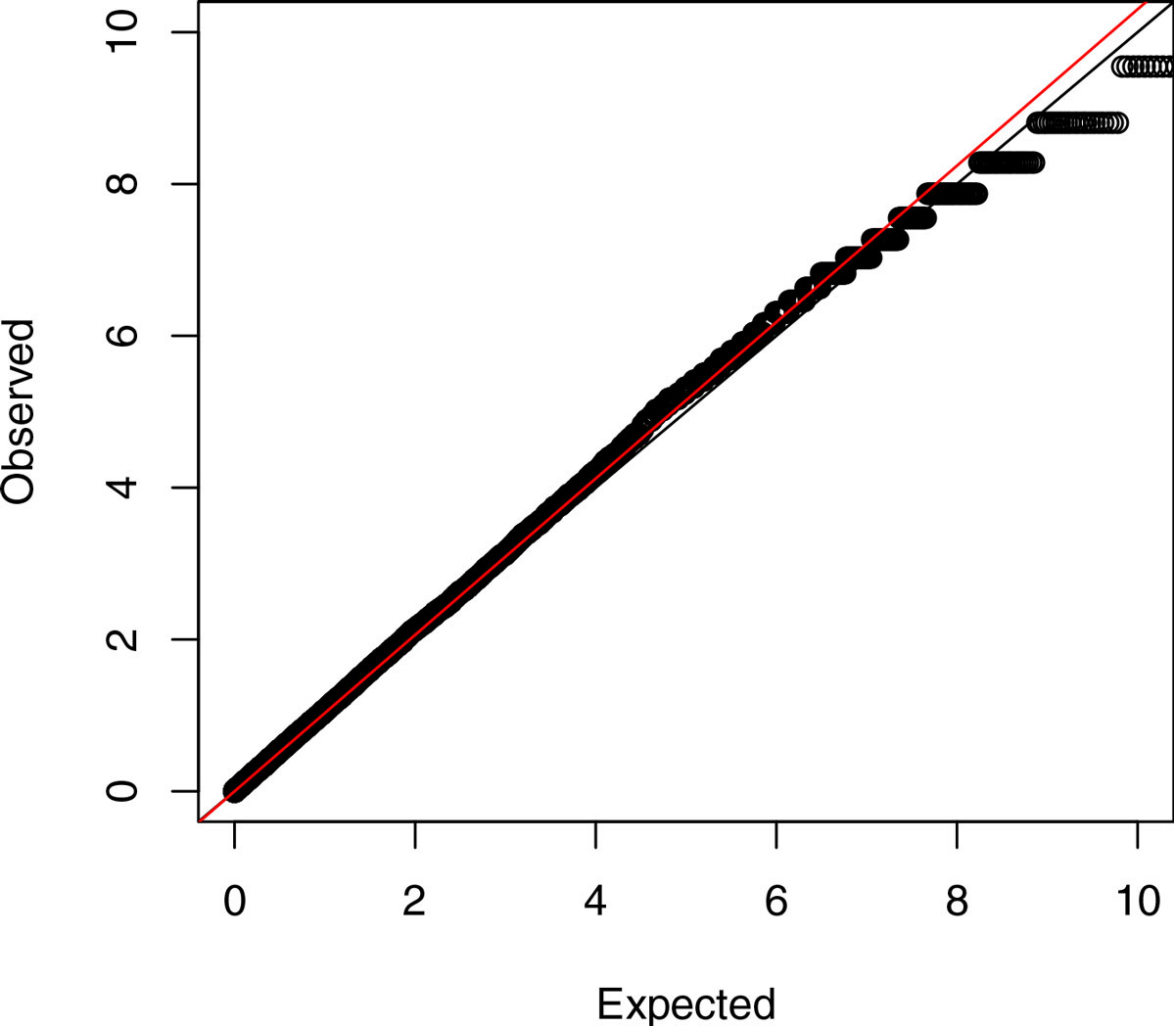
**Quantile-quantile plot for *p*-values of higher criticism (HC)**. *p*-Values of HC for false 10-kbps windows on chromosome 3 were calculated; rare variants were collapsed by equal-weight sum of genotypes.

We assessed the type I error control for HC under various grouping and collapsing strategies. Figure 1 shows the quantile-quantile plots for HC *p*-values of false windows on chromosome 3 based on 10-kbps fixed windows for grouping variants. Certainly, the type I error is well controlled because the observed *p*-values are close to the expectation and the values of genomic inflation factor *λ*[12] is near 1. We also evaluated the type I error rate control under 100- and 500-k windows, gene-based grouping, different MAF thresholds for defining rare variants (0.01 or 0.05), and with equal or MB weight in the collapsing process. Results indicate good type I error rate control (not shown here because of limited space).

**Figure 2:**
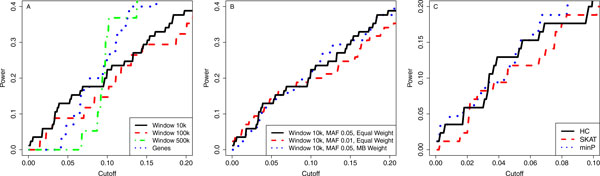
**Power of higher criticism (HC) for detecting all true windows on chromosome 3**. (A) Comparison among variant-grouping strategies by fixed windows (10, 100, and 500 kbps) and genes. (B) Comparison among minor allele frequency thresholds for rare variants and weighting strategies for collapsing rare variants. (C) Comparison among three association tests. ***MB*, **Madsen-Browning; ***minP*, **minimum ***p***-value method; ***SKAT*, **sequence kernel association test.

We compared the power of HC test for detecting true association windows on chromosome 3 under different grouping and collapsing strategies. The left panel in Figure 2 shows the power curves based on three sizes of fixed windows and genes with equal weight collapsing of rare variants. For large *p*-value cutoffs, bigger windows have higher power. However, the 10-kbps window is preferred when the *p*-value cutoff is less than 0.1, where it is of more practical interest for controlling type I errors appropriately. The middle panel in Figure 2 shows the comparison for power of detecting all true 10-kbps windows under different MAF thresholds for defining rare variants and different weighting scheme for collapsing rare variants. The smaller threshold 0.01 helps to get an improvement of the power when the *p*-value cutoff is less than 0.05. It seems the MB weight does not help much.

We compared the power of the above association tests for detecting all true 10-kbps windows on chromosome 3, with equal-weight collapsing and MAF threshold 0.05 for rare variants. From the right panel of Figure 2, HC and minP are similar in the range of small *p*-values. However, minP is not good for weak effects because its power curve drops quickly for larger *p*-value cut-offs. Our power cure is essentially a receiver operating characteristic curve if a full range of cutoffs in (0, 1) is used. Regarding to the area under curve (AUC), HC has the largest value (0.6555) followed by SKAT (0.6496) and minP (0.6327).

**Figure 3:**
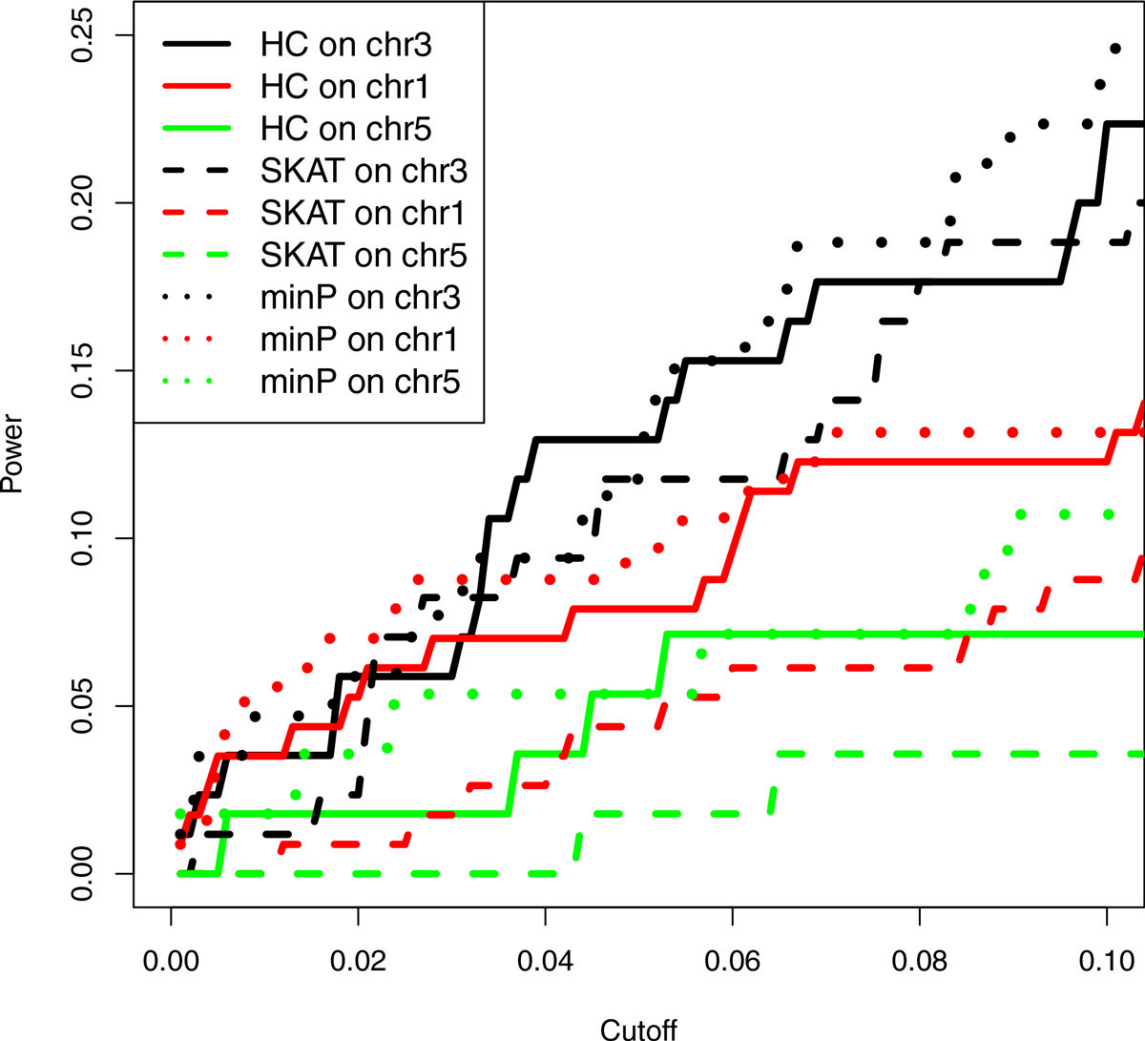
**Power for detecting all true windows on chromosomes (chr) 1, 3, and 5**. Window size was 10 kbps with equal weight for variants. ***minP*, **minimum ***p***-value method; ***SKAT*, **sequence kernel association test.

Figure 3 shows the power of minP, HC, and SKAT for detecting all true windows on chromosomes 1, 3, and 5 (with 10-kpbs windows, equal-weight collapsing, and MAF 0.05 for rare variants). HC and minP have their own advantages for different chromosomes at different cutoffs, but HC performs consistently better than SKAT. However, the power is low in general. Because HC is supposed to be optimal for weak and sparse signals (at least based on asymptotic argument) [6], the low-power phenomenon likely indicates that the sample size (142 independent individuals) is still too small for statistical association studies to detect weak genetic effects by WGS data.

**Figure 4:**
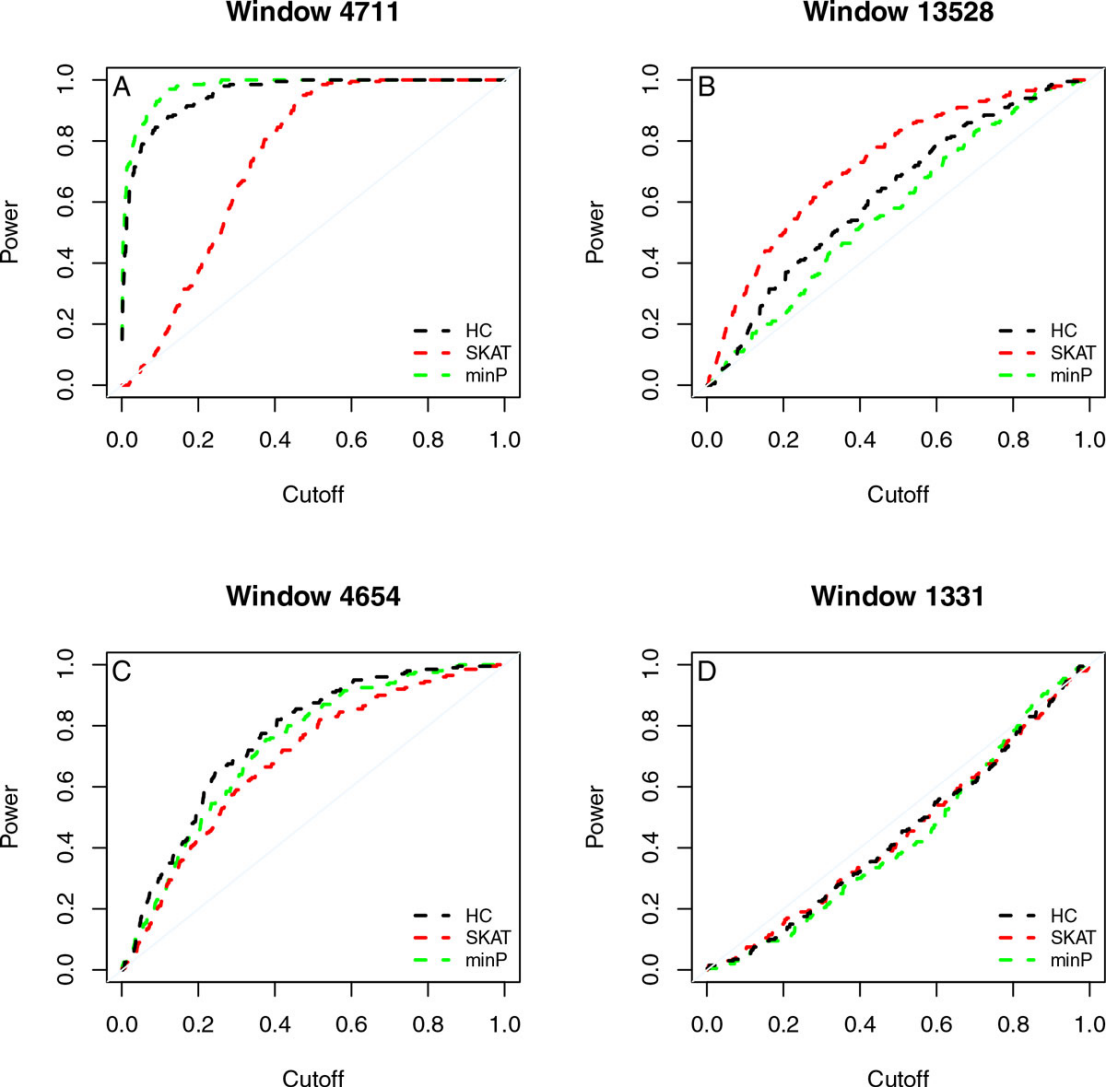
**Comparison of power for detecting specific windows among three association tests**. Power of detecting a specific window over 200 simulation replicates. Window size was 10 kbps with equal weight for collapsing rare variants. (A) Minimum ***p***-value method (minP) is the best. (B) Sequence kernel association test (SKAT) is the best. (C) Higher criticism (HC) is the best. (D) No power for any tests.

To study the capabilities of the association tests in detecting specific signal patterns, we studied their power (i.e., true positive rates) in detecting specific windows over 200 simulation replicates. As illustrated in Figure 4, there are four representative patterns of the comparisons for the 85 true windows on chromosome 3. In particular, 15 windows have HC more powerful than SKAT (left two panels), 10 windows have SKAT more powerful than HC (upper right panel), and 60 windows have almost no power for any tests (the lower right panel). For each comparison pattern, we checked the signal patterns of the corresponding windows. Roughly speaking, minP is likely the most powerful if the windows contain a strong true variant; SKAT is likely the best when the windows contain more but weaker true variants; HC is likely the best when the windows contain fewer and weaker true variants. It is interesting to see that when minP is the best, HC is better than SKAT; when SKAT is the best, HC is also better than minP. Thus, HC has a balanced performance for both strong and weak effects. For all windows, the type I error rates of HC and minP were well controlled (results are available on request).

## Discussion

We compared the HC and SKAT at a "plain" setup with equal weighting scheme and fixed windows, with no bias in choosing variants engaged into the test procedure. This helps us to understand the statistical foundation of these two methods for fair comparison. HC is likely preferred for weak and sparse signals and did show stronger performance in this scenario according to the WGS data and the GAW18 simulations. On the other hand, both methods have great potential to improve the power through, for example, including environmental factors as covariates into the testing.

Several future works could be considered based on the limitations of the current study. First, it would be nice to further confirm the patterns of comparisons among these association methods by simulated and real data with much larger sample size. Second, asymptotic distribution or larger permutations are needed for HC to obtain more accurate *p*-values for genome-wide type I error control. Third, more sophisticated collapsing strategies should be studied based on prior information of genetic architectures. For example, we can consider weighting based variants' apparent effect sizes [13] or data-driven weights [14]. We can also focus on the effects from a subset of meaningful variants from protein-coding point of view [15] or according to evolutionary conservation and functional effects [16]. Last, because the HC procedure is a *p*-value combination approach, it can be flexibly applied to broader context, such as for family data analysis based on mixed effect models or for meta-analysis, which combines different data resources or research results.

## Conclusions

We proposed a framework to apply the HC approach to WGS data. Our analysis of GAW18 simulation data shows that the accurate type I error control by HC is not affected by various grouping and collapsing strategies for rare variants. Our results show that smaller grouping windows (e.g., 10 kbps) are preferred over larger windows or gene-based grouping. For collapsing rare variants at a type I error rate less than 0.05, the MAF threshold of 0.01 is superior to 0.05, and a MB-weighted sum does not provide improvement over an equal-weighted sum. HC likely performs better than SKAT and the minP method in detecting weak and sparse genetic effects.

## Competing interests

The authors declare that they have no competing interests.

## Authors' contributions

ZW designed the overall study. JX, LY and ZW conducted statistical analyses and drafted, read, and approved the final manuscript.
